# Examining the relationship between face processing and social interaction behavior in children with and without autism spectrum disorder

**DOI:** 10.1186/1866-1955-6-35

**Published:** 2014-08-29

**Authors:** Blythe A Corbett, Cassandra Newsom, Alexandra P Key, Lydia R Qualls, E Kale Edmiston

**Affiliations:** 1 Department of Psychiatry, Vanderbilt University, PMB 40, 230 Appleton Place, Nashville, TN 37203, USA; 2 Vanderbilt Kennedy Center for Research on Human Development, Vanderbilt University, PMB 40, 230 Appleton Place, Nashville, TN 37203, USA; 3 Department of Psychology, Vanderbilt University, PMB 40, 230 Appleton Place, Nashville, TN 37203, USA; 4 Department of Pediatrics, Vanderbilt University, PMB 40, 230 Appleton Place, Nashville, TN 37203, USA; 5 Department of Hearing and Speech Sciences, Vanderbilt University, PMB 40, 230 Appleton Place, Nashville, TN 37203, USA; 6 Vanderbilt Brain Institute, Vanderbilt University, PMB 40, 230 Appleton Place, Nashville, TN 37203, USA

**Keywords:** Autism spectrum disorder, Play, Ecological validity, Neuropsychology, Face memory

## Abstract

**Background:**

Children with autism spectrum disorder (ASD) show impairment in reciprocal social communication, which includes deficits in social cognition and behavior. Since social cognition and social behavior are considered to be interdependent, it is valuable to examine social processes on multiple levels of analysis. Neuropsychological measures of face processing often reveal deficits in social cognition in ASD including the ability to identify and remember facial information. However, the extent to which neuropsychological measures are associated with or predictive of real-world social behavior is unclear.

**Methods:**

The study investigated 66 children (ASD 34, typically developing (TD) 32) using neuropsychological measures of face processing (identity, affect, and memory). Children also participated in a peer interaction paradigm, which allowed observation and coding of natural social interaction behaviors during play with peers (e.g., Self-Play, Cooperative Play, Verbal Bout). ANCOVA, regression, and correlation models analyzed between-group differences, the ability of neuropsychological measures to predict social behavior, and the strength of the associations.

**Results:**

Between-group differences were shown on Memory for Faces Delayed and the peer interaction variables Self-Play and Verbal Bout. Regression models indicated that Memory for Faces Delayed predicted the amount of Self-Play, Equipment use alone, and Cooperative Play with peers on the playground. Autism symptomology only predicted verbal exchange with peers.

**Conclusions:**

Face memory strongly predicts relevant social engagement patterns in both children with and without ASD. Impairment in facial memory is associated with reduced ‘real-world’ social interaction and more self-play, whereas higher performance in face memory predicts more cooperative play. Results highlight the strong connection between face memory and reciprocal social interaction, suggesting that improvement in one may benefit the other.

## Background

Social behavior relies on a complex neural network of cognitive, emotional, and behavioral systems in which perception and behavior are causally linked [1] and highly influenced by developmental and compensatory factors [2]. Social cognition is the ability to recognize, manipulate, and respond to socially relevant information, thereby creating complex representations of self and others that can be used flexibly to guide behavior [1]. Kennedy and Adolphs [2] succinctly describe four interdependent levels of analysis including the social *brain* that operates social *cognition* that in turn produces social *behavior*, which when integrated over time and context, establishes social *functioning*. Therefore, the study of social impairment, especially in a disorder marked by primary deficits in social communication such as autism spectrum disorder (ASD) [3], necessitates examination across these levels.

Autism spectrum disorder is characterized by measurable deficits in social cognition (e.g., [4]), behavior [5], and everyday functioning [6] with converging evidence of dysfunction in social brain networks (e.g., [7-9]). The quality and quantity of reciprocal social interaction in children with ASD is significantly impaired. It is well established that children with ASD initiate fewer social interactions and receive fewer social overtures from other children [10]. When they do engage with peers, children with ASD often show heightened stress evidenced by increase in salivary cortisol when playing with peers, especially as they get older [5,11,12]. Importantly, children with ASD who engage in reciprocal social interaction show greater participation in social and recreational activities, inclusion while in school, and more independence in activities of daily living, all areas in which children with ASD often struggle [13].

There has been substantial speculation as to the contributory factors leading to the social interaction deficits in ASD. Consistent with the aforementioned components outlined by Adolphs [1], the theoretical frameworks typically emphasize either perceptual [14], cognitive [4], or motivational [15] factors that give rise to the significant impairment in reciprocal social interaction or functioning. It is likely that different triggers and trajectories lead to the same challenging social outcome. Collectively, these theories support the social framework articulated by Kennedy and Adolphs [2]. If this is the case in ASD, then associations across social levels should be observable and informative. For example, the measurement of social cognition should predict behavior.

Social cognition requires developing expertise to recognize faces of conspecifics [16]. While a deficit in face processing in ASD is not pathognomonic (meaning that it is not a definitive diagnostic sign), many children show significant impairment in the identification and memory for facial information. In fact, some researchers have characterized poor social processing of facial information as an autism endophenotype [17,18]. However, not all aspects of face processing are qualitatively or quantitatively impaired [19].

There is extensive evidence of atypical face perception processes in infants, children, and adolescents with autism. Poor reciprocal eye contact and gaze aversion have been documented in 1-year-olds later diagnosed with autism (e.g., [14]) and in 3- to 13-year-olds with ASD [20]. Nine-month-old infants at high risk for ASD showed atypical brain responses to familiar vs. novel faces [21], while toddlers (18–30 months) with ASD evidencing lower social and verbal skills exhibited a slower pattern of learning (habituation to) facial information than comparison children [18]. Langdell [22] performed one of the first studies of face recognition in autism and found that 8- to 10-year-old children with ASD were better at identifying peers from the lower half of the face than typically developing (TD) peers but were worse at recognizing the face from the upper half. Klin et al. [23] investigated a large sample of children (mean age 7 years) with autism, pervasive developmental disorder-NOS, and non-PDD (intellectual disability and language disorders) revealing profound deficits in face recognition exclusively in the autism group that could not be explained by cognitive level or demands of the task. More recent evidence indicates that older children with ASD (8- to 14-year-olds) are less effective than typically developing peers at recognizing repeatedly presented faces, suggesting reduced ability to remember faces when not explicitly instructed to do so [24].

Affect recognition is also a key component of social cognition and there is some evidence of differences in ASD [25-27]. For example, Kuusikko and colleagues [28] found that children with ASD had poorer performance on an emotion recognition test compared to TD children and often misconstrued more ambiguous stimuli as showing negative emotions. Krebs and colleagues [29] also showed differences in facial and emotion processing which the authors speculate may be due to children with ASD processing face identity and affect separately, whereas TD children process them simultaneously. In a study of facial scanning, individuals with ASD showed impaired performance in emotion recognition primarily for fear [30]. Grossman and colleagues [31] reported that children with Asperger syndrome did not differ from typical controls in the recognition of simple emotions yet exhibited qualitative differences on more demanding tasks, suggesting the need for compensatory strategies in processing facial affect. Other studies have not found impaired affect processing in ASD and attribute differences observed across previous studies to task demands (e.g., [17,32,33]). Taken together, facial affect recognition deficits are not consistently found in individuals with ASD and some face processing abilities may be comparable to typically developing peers.

A recent comprehensive review of face perception and memory [19] concluded that many individuals with ASD are able to identify faces utilizing face-specific perceptual mechanisms; however, they consistently show difficulty in remembering facial information, especially on measures involving a delay. For example, Boucher and Lewis [34] compared memory for faces and for houses and showed that children with ASD (mean age 9 years) had worse performance on memory for faces, but memory for houses was comparable across the groups. In a study of children 7 to 12 years of age, Hauck et al. [35] found a memory-specific deficit for faces in children with ASD amidst similar matching skills when compared to TD children. Memory for faces as it relates to other areas of functioning has been infrequently studied. Recently, it was shown that better face memory in adolescents with ASD was associated with fewer characteristics of autism [36].

The aforementioned neuropsychological studies collectively indicate that children with ASD often demonstrate impairment in processing facial information. Yet, the extent to which these deficits are predictive of everyday social functioning is unclear. Characterizing the autism phenotype warrants not only enhanced understanding of underlying processes of social functioning but also the inclusion of methods that more adequately approximate the naturalistic demands of complex social situations [37]. Neuropsychological measures are used to establish baseline functioning, identify areas of strength and weakness, document change in status, and guide treatment [38]. Such measures are intended to reflect an individual's everyday functioning and to predict outcomes [39-41]. Recently, the ecological validity of such measures, especially in pediatric populations, has been called into question [42].

Ecological validity in neuropsychology refers to the functional and predictive relationship between an individual's neuropsychological performance on a test and their behavior in a variety of real-world settings [43]. Specifically, ecological validity consists of verisimilitude (extent to which cognitive demands of lab tests resemble everyday demands) and the veridicality (extent to which performance on the given measure is predictive of day-to-day functioning) of a measure [39,44-46]. The majority of the research on the ecological validity of neuropsychological measures has been conducted on adults; however, there is critical need to carry out pediatric studies that inform the complex nature of the cognitive, social, and emotional development of children [42]. Additionally, Sbordone and Ruff [47] stressed the need to corroborate neuropsychological findings by also observing individuals in real-world settings. In terms of specific domains of functioning, the use of simple social perception tasks, such as the pictorial representations of conspecifics, as representative of complex social behavior has been similarly questioned [48]. In other words, to what extent does viewing pictures of facial stimuli, frequently employed in human research, represent more complex social behavior?

Since real-world social interactions are inherently complex and variable, it is challenging to recreate them in standardized instruments. This challenge can be addressed by examining component parts, which is often an objective in neuropsychological testing. For example, social interaction is comprised of many facets (e.g., face memory, affect recognition) and the component parts may not map explicitly onto the real-life behavior. The goal, nonetheless, is to identify the parts that have predictive value.

The current study aimed to address some of these concerns by examining the ability of neuropsychological measures of social cognition (affect recognition, face identification, and face memory) to predict social communication with peers in a naturalistic playground setting. Using a mixed method approach, the study assessed the extent to which neuropsychological measures reflect the social communication profiles of children with and without ASD during play. In the process, we test the social interdependent model [2] and hypothesized that face-processing ability would be associated with social interaction behaviors during play with peers.

## Methods

### Participants

The study sample consisted of 66 un-medicated, male children between 8 and 12 years, 34 with ASD (mean age = 10.03 years) and 32 with typical development (mean age = 9.62 years). ASD diagnosis was based on the Diagnostic and Statistical Manual (DSM-IV) criteria [49] and established by (1) a previous diagnosis by a psychologist, psychiatrist, or behavioral pediatrician with ASD expertise; (2) current clinical judgment (BAC or CN); and (3) corroborated by the Autism Diagnostic Observation Schedule (ADOS) [50], administered by research-reliable personnel, with a total score at or above the ASD threshold for Module 3. All participants were prepubertal based on parent report on the Pubertal Development Scale [51].

The Vanderbilt University Institutional Review Board approved the study. Informed written consent was obtained from parents, and verbal and written assent was obtained from child participants prior to inclusion in the study.

Participation in the study required two visits to the University. During visit 1, the diagnostic and neuropsychological measures were administered, and during visit 2, the participants completed the peer interaction paradigm. The visits occurred within a 1-month period, and the second visit was always conducted in the afternoon between 2:00 and 5:00 pm.

### Diagnostic and inclusion variables

#### Autism Diagnostic Observation Schedule

The ADOS [50] is a semi-structured interview designed to assess behaviors characteristic of ASD. A score of 8 or greater on the social-communication domain of the ADOS Module 3 was required for inclusion in the ASD group. The mean score (and standard deviation) for Social Communication was 12.67 (3.89), Repetitive Behavior was 2.58 (1.54), and the average total score was 12.58 (3.92).

#### Wechsler Abbreviated Scale of Intelligence

The Wechsler Abbreviated Scale of Intelligence (WASI) [52] is a measure of cognitive ability used to obtain an estimate of intellectual functioning. Inclusion in the study required an estimated IQ of 70 or higher. The average estimated IQ was 100.62 (18.56) for children with ASD and 118.06 (13.79) for TD peers, *t*(1,65) = 4.47, *p* = 0.001. The scores ranged from 72 to 145.

#### Social Communication Questionnaire

The Social Communication Questionnaire (SCQ) [53] was used as a screening tool for ASD (scores ≥15 are suggestive of ASD, while scores ≥22 are suggestive of autism). The exclusion criterion for a typically developing child was a score ≥10; however, no participants were excluded based on this criterion. The mean SCQ scores were 21.26 (6.92) for the ASD group, and 2.52 (2.02) for the TD group, *t*(1,67) = −14.96, *p* = 0.001.

### Neuropsychological variables

Three Developmental NEuroPSYchological Assessment (NEPSY) [54] subtests *Affect Recognition*, *Memory for Faces Immediate*, and *Memory for Faces Delayed* were administered to assess social perception (recognizing emotions and identifying faces, respectively). Affect recognition requires the child to recognize affect from pictures of children's faces (happy, sad, fear, anger, neutral, disgust). Memory for Faces Immediate is a face recognition task that requires the child to select previously seen children's faces among three choices following a brief 5-s initial exposure. The Memory for Faces Delayed requires the child to again choose the previously viewed faces after a 30-min delay. The NEPSY has been used in studies of children with ASD [55-57]. The scaled scores from the subtests were used as predictor variables.

### Peer interaction playground paradigm

**Figure 1:**
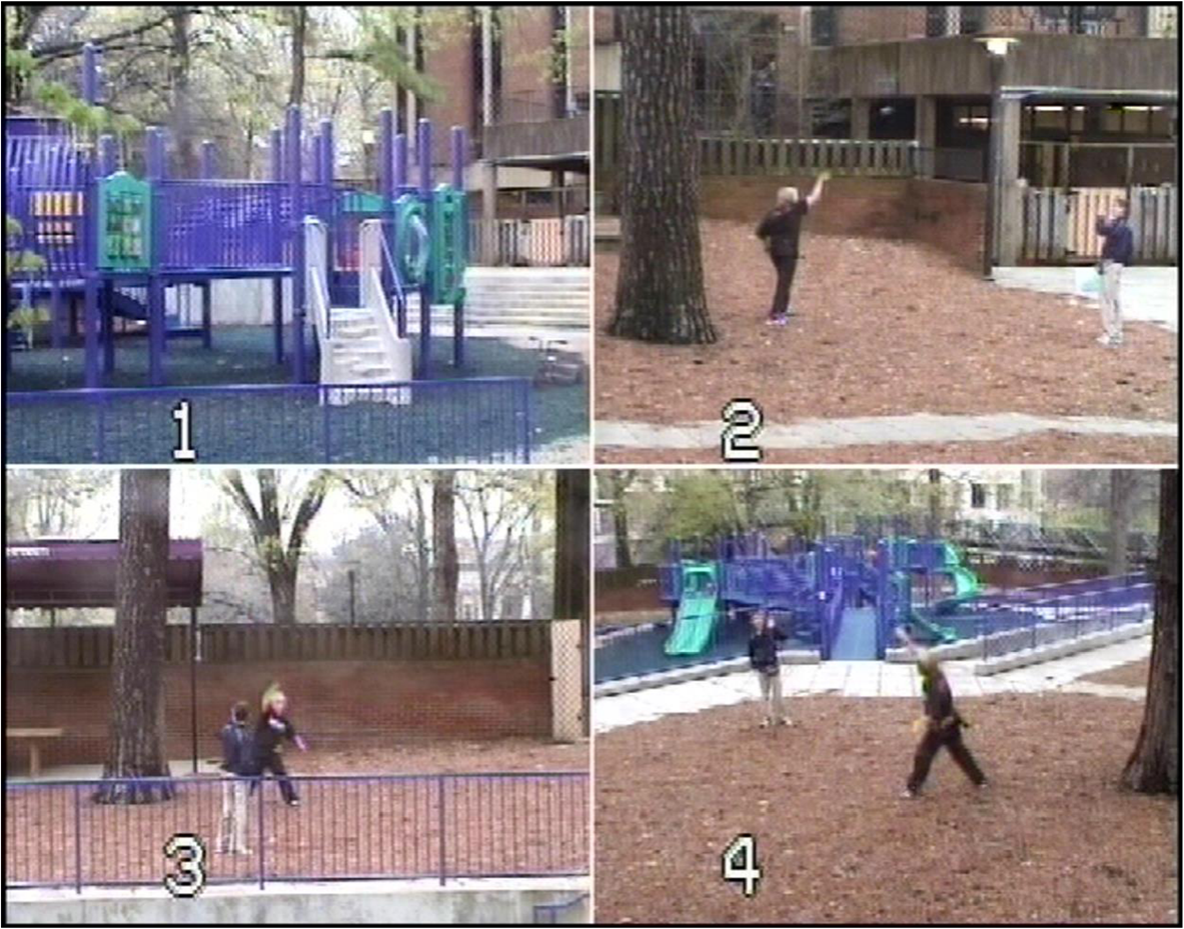
**Image of the Playground Paradigm with four camera views (numbers 1 to 4).** The study took place on a 130 by 120 ft fenced-in playground containing large equipment, swings, walkways and open space for interactive games. Research personnel remained in the building while monitoring the protocol from within the behavioral lab, allowing the participants to engage in more natural play behavior. Interactions were recorded using video and audio equipment.

The Peer Interaction Paradigm was developed to examine social exchanges within a playground environment occurring between children with and without autism [5]. The 20-min paradigm incorporates periods of free play and opportunities for cooperative play that are facilitated by a typically developing confederate child of the same age and gender. The 130 by 120 ft fenced-in playground is attached to a Vanderbilt University preschool and contains large equipment, swings, walkways, and open space for interactive games. For the duration of the protocol, adult research personnel remained in the building while monitoring ongoing activity from within the behavioral lab, allowing the participants to engage in more natural play behavior.Interactions were video recorded using four professional 70 Sony PTZ (New York, NY, USA) remotely operated cameras housed in glass cases and affixed to the four corners of the external fence of the playground (see Figure 1). The cameras contain pan, tilt, and zoom features allowing full capture of the playground. Remote audio communication was established by Sennheiser body pack (Old Lyme, CT, USA) and Audio-Technica transmitters and receivers (Stow, OH, USA), which functioned as battery-operated microphones that were clipped to the shirt of each child and simultaneously recorded by an eight-channel mixing board.

Each interaction paradigm involved three children, a child with ASD, a TD child, and a confederate of the same age and gender. The confederate provided behavioral structure to the play by permitting key interactive sequences to occur within an otherwise natural interaction and setting. Moreover, the confederate maintained an even level of play to prevent increased aerobic activity, which could affect cortisol levels. The trained confederate solicited play simultaneously from the two research participants following a cue provided by research personnel through an earpiece with a remote transmitter.

The paradigm was divided into four 5-min time (T) periods of intermittent free play and solicited play. The first period (T1) consisted of unsolicited free play. During the second period (T2), the confederate solicited interaction on the play equipment for cooperative play. During the third period (T3), the confederate was instructed to again engage in free play. During the fourth period (T4), the confederate solicited the two participants to engage in a cooperative game involving toys. Based on our previous results [11] and in order to predict social exchange with peers, this latter period when the children are invited to engage in reciprocal social interaction with the confederate is the primary time period of interest.

### Behavior coding

The Observer XT Version 8.0 software [58] was used for the collection and analysis of the interaction observational data. Data were analyzed using our established protocol [5,11,12]. The paradigm utilizes a transactional approach in which ‘bouts’ of engagement initiated by one participant set the stage for a sequence of behaviors occurring between two or more children [59].

### Behavioral variables

#### Playground behaviors

Social and communication playground behaviors included duration variables. Specifically, percentage of time engaging in social behaviors were calculated for each behavior: Verbal bout (reciprocal verbal exchange between two or more children), Cooperative Play (reciprocal engagement of play in a collaborative game), Self-Play (independent play with a toy or object alone but in the presence of others), Equipment Play with Self (playing on structured play equipment alone), and Equipment Play with Group (playing on structure play equipment between two or more children) for each of the four time periods. The peer interaction variables ranged from 0% to 100% duration in which the participant was engaged in the specific behavior. Inter-rater reliability was calculated for a random sample of 25% of observations. Observer [58] reliability calculations for the specific behaviors were cooperative play 91% and *k* = 0.89, verbal bout 90% and *k* = 0.85, use of equipment 87% and *k* = 0.74, self-play 90% and *k* = 0.85.

### Statistical analysis

All statistical analyses were conducted using SPSS Software, Version 21.0 (SPSS Inc., Chicago, IL, USA). The distribution of the variables was examined and the range of scores presented as minimum and maximum are reported in the description of the variables (i.e., IQ, SCQ). Skewness and kurtosis were examined to achieve a distribution between −1.0 and 1.0 (e.g., Memory for Faces 0.009 and − .38 and Memory for Faces Delayed −0.51 and − .33, respectively).

Using univariate analysis of covariance (ANCOVA), between-group differences were calculated with IQ included as a covariate for the social neuropsychological measures (Affect Recognition, Memory for Faces Immediate, and Memory for Faces Delayed) (see Table 1). Additionally, ANCOVA models were conducted to determine between-group differences in playground behaviors (Self-Play, Cooperative Play, Equipment Play Self, Equipment Play Group, and Verbal Bout), previously shown to discriminate the groups during solicited play by the confederate [5,11,12]. Skewness and kurtosis for behavioral variables were Self-Play 1.45, 1.16; Cooperative Play −1.0, −.144; Equipment Play 1.54, 1.57; Equipment Play Group −1.0, −.07; and Verbal Bout −1.7, 1.78, respectively. Due to the distribution results, bootstrap simulations for 1,000 samples were conducted at the 95% confidence interval for the playground variables.

**Table 1:** Social communication behaviors between the groups during solicited play

**Percentage of behavior**	**Group**		***df***	***F***	***p***	***η***_**p**_^***2***^
	**ASD**	**TYP**				
Self-play	25.49	10.01	2, 63	4.3	0.04	.07
	(30.52)	(20.01)				
Cooperative Play	62.44	80.97	2, 63	3.08	0.08	.05
	(35.67)	(21.41)				
Equipment Play Self	24.41	13.00	2, 63	1.24	0.27	.02
	(28.99)	(24.15)				
Equipment Play Group	63.44	78.20	2, 63	2.18	0.15	.04
	(35.15)	(21.87)				
Verbal Bout	73.14	90.63	2, 63	5.66	0.02	.09
	(34.87)	(16.47)				

*ASD* autism spectrum disorder, *TD* typically developing, *F* F statistic, *p p* value, *η*_p_^2^ partial eta squared. The ANCOVA model covaried for IQ.

Linear regression models were used to examine the extent to which neuropsychological (face processing) and diagnostic measures predict social communication between peers on the playground. NEPSY face (affect, identity, memory) variables, diagnosis, and symptom severity (SCQ) were entered into the model to predict the social communication behaviors with peers. For each predictor in the regression equation to control for multiplicity effects across the five behavioral variables, we used the Bonferroni-Holm method [60]. This step down procedure is similar to a classical Bonferroni procedure but differs in that a sequential set of tests are conducted that involve layered adjustments of alpha levels based on the number of elements remaining in the original set. The Bonferroni-Holm provides adequate control over family wise error rates but has better power than the classical Bonferroni test (e.g., [61]). Finally, bivariate correlations (Pearson product-moment) were conducted to examine the strength of the associations across the aforementioned variables.

## Results

Based on the above data, the following results were obtained:

The demographic information pertaining to age, diagnostic, social communication and cognitive functioning is presented with the participant and the test measure sections above. Regarding neuropsychological social behaviors, there were significant between-group differences for Memory for Faces Delayed and a trend (*p* = 0.05) for Memory for Faces Immediate (see Table 2). However, there were no significant differences between the groups for Affect Recognition.

**Table 2:** Group differences from neuropsychological measures of face affect, identify and memory scaled scores

**Variable**	**Group**		***df***	***F***	***p***	***η***_**p**_^**2**^
NEPSY Social Perception	ASD mean (SD)	TD mean (SD)				
Affect Recognition	9.06 (2.90)	11.09 (2.18)	2, 62	2.81	0.09	.04
Memory for Faces Immediate	7.56 (3.27)	10.39 (3.3)	2, 62	3.99	0.05	.06
Memory for Faces Delayed	8.88 (3.54)	11.97 (2.43)	2, 62	6.87	0.01	.14

*ASD* autism spectrum disorder, *TD* typically developing, *F F* statistic, *p p* value, *η*_p_^2^ partial eta squared. The ANCOVA model covaried for IQ. Average scaled scores range from 7 to 13 [54].

The playground behavior results are presented in Table 1. Significant differences were observed between ASD and TD children for Self-Play and Verbal Bouts. Based on linear regression, the social behavior variables Affect Recognition, Memory for Faces Immediate, Memory for Faces Delayed, and Diagnosis and the SCQ were entered into the model as predictors of social behavior. The results are presented in Table 3 and indicate that Memory for Faces Delayed was a significant predictor of Self-Play and Equipment Play Alone. The Memory for Faces Delayed was the only significant predictor of the amount of Cooperative Play. The SCQ was the only predictor of Verbal Bout, although the overall regression model was at trend level significance. In summary, memory for faces following a delay strongly predicted several of the social behavior variables, including equipment play (as a group and alone), self-play, and cooperative play.

**Table 3:** Affect recognition and memory for faces as predictors of playground behavior

**Behavior**	**Memory for Faces Delayed**				**Memory for Faces Immediate**				**Affect Recognition**				**Regression Model**		
	***t***	***β***	***p***	**Boot *p***	***t***	***β***	***p***	**Boot *p***	***t***	***β***	***p***	**Boot *p***	***F***	***p***	***R***^**2**^
Self-Play	−3.01	−0.61	0.01**	0.01**	1.76	0.33	0.09	0.05	0.07	0.11	0.94	0.94	3.01	0.02*	0.24
Coop Play	2.98	0.59	0.01**	0.01**	−1.46	−0.27	0.15	0.11	−0.19	−0.03	0.85	0.85	3.26	0.01**	0.25
Equip Play A	−3.35	−0.68	0.02*	0.02*	1.95	0.37	0.06	0.04	0.92	0.14	0.36	0.31	2.60	0.04*	0.21
Equip Play G	2.48	0.52	0.02	0.02*	−1.05	−0.20	0.30	0.38	0.25	0.04	0.80	0.79	2.17	0.07	0.18
Verbal Bout	1.25	0.26	0.22	0.25	−0.85	−0.16	0.39	0.41	−1.01	−0.16	0.32	0.14	2.32	0.06	0.19

Results from primary aims are presented. Due to violation of normal distribution, bootstrap resampling was used and results are presented based on 1,000 samples (Boot *p*). For each significant predictor in the regression equation to control for multiplicity effects across the five behavioral variables, we used the Bonferroni-Holm method, a step down procedure in which a sequential set of tests are conducted that involve layered adjustments of alpha levels based on the number of elements remaining in the original set.

*t t* statistic, *β* beta value, *p* corrected alpha levels using Bonferroni-Holm method (*p < 0.05, **p < 0.01), *Coop* cooperative, *Equip* equipment, *A* alone, *G* group.

It is important to note that the SCQ was a significant predictor for only Verbal Bout (F(6,53) = 1.88, *p* = 0.04, *R*^2^ = 0.19), suggesting that autism symptomology influenced the amount of social communication with peers. However, the strongest predictor for the social interaction variables on the playground across the groups was the delayed face memory task.

**Figure 2:**
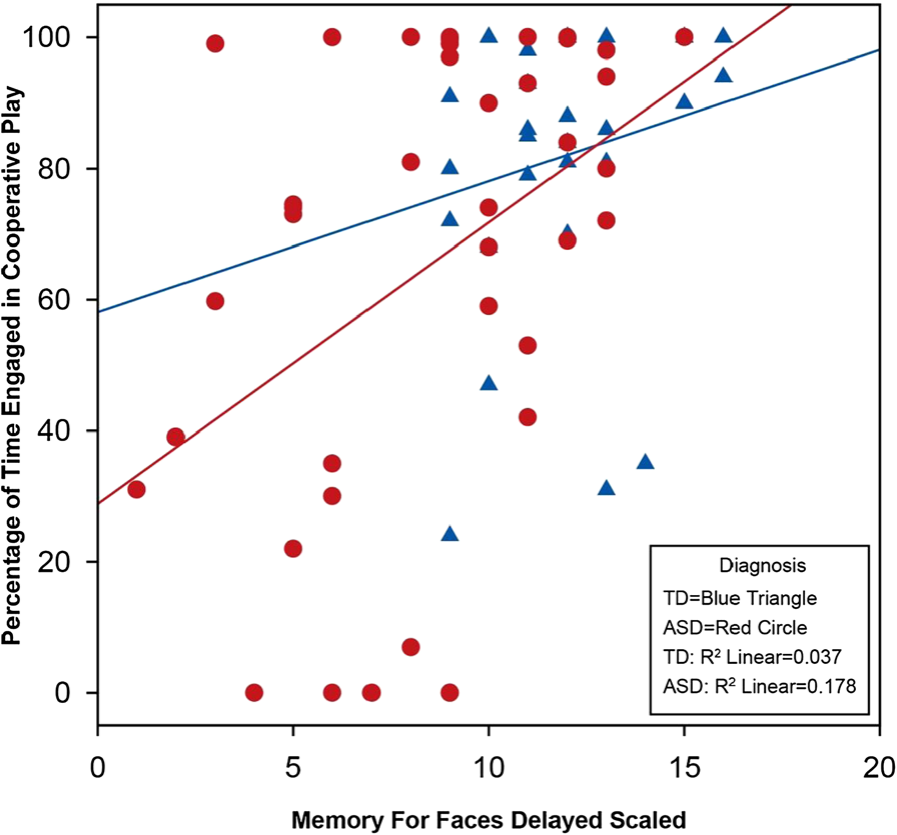
**Scatterplot of associations between Memory for Faces Delayed and Cooperative Play in children with ASD and TD.** The *Y*-axis reflects the percentage of time children with ASD (red circle) and TD (blue triangle) engaged in cooperative play with peers, whereas the *X*-axis reflects the scaled score from the Memory for Faces subtest. The regression lines show a positive correlation between the variables which is most pronounced in the ASD group.

Correlations between the social neuropsychological and playground variables are presented in Table 4. Since diagnosis and IQ were not significant predictors of the association between memory for faces and most of the behavioral variables, the correlational analyses were conducted on the combined sample. The strength of the association ranges from relatively weak (Affect Recognition and Self-Play) to strong (Memory for Faces Delayed and Cooperative Play) (see Figure 2). As expected, there were strong correlations between the face measures, especially between face identification and memory. Additionally, several of the behavioral variables showed strong correlations, such as an inverse relationship between self-play and equipment play with a group. Exploratory partial correlations controlling for diagnosis continued to show significant (*p* < 0.01) strong correlations between Memory for Faces Delayed and Self-play *r* = −.35, Cooperative Play *r* = .38, and Equipment Play with Group *r* = .37.

**Table 4:** Correlations among social study variables

	**Affect Recog**	**MFI**	**MFD**	**Self-Play**	**Coop Play**	**Equip Play G**	**Equip Play A**
Affect Recog	-						
MFI	0.44**	-					
MFD	0.5**	0.73***	-				
Self Play	−0.26*	−0.15	−0.43***	-			
Coop Play	0.29*	0.25*	0.45***	−0.87***	-		
Equip Play Group	0.36**	0.27*	0.43***	−0.77***	0.89***	-	
Equip Play Alone	−0.13	0.1	0.35**	0.77***	−0.84***	−0.74***	-
Verbal Bout	−0.14	0.11	0.26*	−0.74***	0.82***	0.75***	−0.76***

*Affect Recog* Affect Recognition, *MFI* Memory for Faces Immediate, *MFD* Memory for Faces Delayed, *Coop Play* Cooperative Play, *Equip Play G* Equipment Play with Group, *Equip Play A* Equipment Play Alone.

*p < 0.05 level, **p < 0.01 level, ***p < 0.001.

## Discussion

The current study aimed to investigate the complex social profile in children with ASD by examining it at two levels of analysis—social cognition and social behavior. It also evaluated the ecological validity of standardized neuropsychological social measures to predict real-life social interactions with peers in a playground setting.

The investigation is among the first to examine the performance of children on standardized neuropsychological measures, which assess specific types of social perception as predictors of actual social communication with peers in a naturalistic setting. The results show that immediate face recognition and in particular, the delayed component of the face memory task, has strong veridicality by predicting social and play behaviors in the natural setting. Although the use of static pictorial faces as representative of more complex social behavior has been called into question [48] and the playground interactions did not explicitly require memory for faces, the findings support the strong link between the recognition of faces and complex social skills. Moreover, the data show that face memory impairment has real-world implications. The reported strong associations begin to address the growing need to examine the ecological validity of neuropsychological measures and constructs in order to confirm their predictive value in children [42].

Using regression models, the neuropsychological measures Affect Recognition, Memory for Faces Immediate, and Memory for Faces Delayed were compared with the social behaviors on the playground. Memory for Faces Delayed was a strong predictor of social functioning with peers and the only variable to survive Bonferroni-Holm correction. While Memory for Faces Immediate and Memory for Faces Delayed utilize the same stimuli and are temporally connected, they are dissociable as demonstrated by the strong, distinct relationship between the delayed face memory component and the social interaction variables. Specifically, regardless of the autism symptomology, better ability to remember faces was associated with more cooperative play with other children and reduced play alone. Thus, performance on a face memory task strongly predicted social interaction patterns with peers during play for children with and without ASD.

Although the primary aim of the investigation was to evaluate social interaction skills, the influence of diagnosis was a natural exploratory aim. It is intriguing that autism symptomology was a primary predictor for only the percentage of time that children with ASD engaged in verbal exchange with peers. The association illustrates that the fundamental deficits in language and conversational skills seen in children with ASD impact the amount of social communication with peers. The findings suggest that the investigation of receptive and expressive language measures with high ecological validity would be a valuable contribution to elucidating the explicit role of language in social exchange.

In the current study, the strongest predictor for the social interaction variables on the playground was the delayed face memory task. This observed relationship indicates that delayed memory for faces is a basic skill supporting social engagement. As previously noted, social behavior relies on a complex neural network in which perception and behavior are causally linked [1,2]. A social cognitive task that is able to predict real-world behavior is clinically valuable. Such is the case with the NEPSY Memory for Faces Delayed, which measures a basic process common to both ASD and TD, predicts functional behavior in a real-world setting, and therefore is ecologically valid [46]. It may be argued that the association also demonstrates convergent validity, which refers to the degree to which two measures presumed to be theoretically related are in fact related [62]. In the current context, the playground variables are conceptualized as measures of real-world social functioning, and therefore, the term ecological validity is more appropriate for the purpose of this investigation.

While the predictive associations between memory for faces and playground social interactions were independent of the diagnosis, analysis of performance on the individual measures demonstrated that face recognition memory in ASD is compromised. A recent comprehensive review of quantitative studies indicated that persons with autism consistently perform worse than TD individuals on tasks of face perception and face memory [19]. Importantly, tasks that do not contain a memory component often fail to discriminate the groups; yet, when there are even minimal memory demands individuals with ASD show notable deficits. The current findings are similar in that there were significant differences between the groups for the face memory component but only modestly for the face identity task without a delay. Additionally, children with ASD were no different from TD on their ability to distinguish different facial expressions, which has also been an inconsistent finding in the literature [8,28,29] (for a review see [63]). It is possible that the measures of affect recognition used across these studies may not be sensitive enough to detect the subtle impairment seen in some individuals with ASD [64].

The contributing etiological factors for face processing difficulties are mixed and include explanations related to aspects of avoidance, attention, or motivation. Some researchers have proposed that the failure to acquire face expertise arises from avoiding eye contact due to heightened autonomic arousal [20]. Others have suggested that poor facial memory may be the result of fundamental impairment in social orienting stemming from disruption in neural circuitry that primes innate responses [65]. Meanwhile, others suggest a lack of motivation to attend to social stimuli, including facial information [15]. Due to the heterogeneity of the symptom profile and diversity of severity in ASD, it is likely that each of these explanations may hold for a portion of the population.

The current study supports the idea that brain, perception, behavior, and functioning are inter-related [2]. While direct brain measures were not employed, the findings strongly support the idea of connectedness across social levels that contribute to the long-standing deficits in ASD especially reciprocal social interaction with peers. Although all the connections were not explicitly studied, the strong correlations across the perceptual and behavioral measures support the bi-directionality of social phenomena. In consideration of treatment, the interconnectedness suggests that improvement in social cognition may improve behavior and vice versa. Indeed, while face memory in ASD is consistently found to be poor, such a deficit is not intractable to treatment and may improve with social skills intervention. Recently, significant improvement in face memory along with reciprocal gains in social and adaptive functioning was reported following a theatre-based intervention targeting social interaction skills in youth with ASD [55,66].

Among the strengths of the current study is the use of the peer interaction paradigm developed to carefully study children in their natural environment while engaging in what children often do best—play, an activity with which many children with ASD struggle. When compared to TD peers, children with ASD demonstrate impaired ability in the quantity and the quality of play [5,12]. While children with ASD have significant difficulty interacting with peers and show diminished interest in engaging with others, there is notable variability in presentation such that social subtypes have been proposed [67]. Previous studies using the paradigm have found significant variability in behavioral responses within the ASD group supporting this notion of social phenotypes [5,11,12]. The utilization of this novel, ecologically valid protocol is a strength of the investigation. The remote control video and audio recording equipment allowed the natural study of play without a contrived environment or forced interaction. Additionally, the gender- and age-matched confederate provided a realistic flow to the interaction. Finally, the study included a relatively large sample of well-characterized, unmedicated children.

Despite these strengths, the following limitations and future directions are acknowledged. The playground protocol included three children, which may be optimal for the study of play in ASD and yet may not fully translate to a school playground setting with many more children. Our enrollment criteria included high-functioning children with ASD, which resulted in a sample with average cognitive functioning. However, we did not match on IQ, and the typically developing group had slightly higher IQ. Nevertheless, we controlled for IQ in the analyses and, as noted above, IQ had no effect on the observed associations between the scores on neuropsychological assessments and real-life social interactions. Additionally, there were significant differences in face memory and play behaviors between the groups; however, the partial eta square effect sizes are rather small, suggesting that other essential factors need to be considered. Importantly, the beta values from the regression models are very strong [68], supporting the primary aim of the study, which was to reveal the predictability and strength of the associations between neuropsychological measures and real-world social functioning despite limited diagnostic differences. The degree and strength of the association between facial memory and social behavior in adolescent and adult populations are also unclear and beyond the scope of the current investigation. Finally, these data also do not provide objective guidance as to etiological factors that may underlie face-processing deficits in ASD.

Based on the cumulative evidence and findings herein, the following future directions are proposed. Social functioning is diverse and complex; therefore, examinations beyond the contributions of face memory in predicting social functioning are clearly warranted. The development of measures and protocols that approximate the complexity and flexibility necessary for adequate social functioning are also needed. While ASD and the phenotypic expression are heterogeneous, deficits in face processing are inextricably linked to the higher demands of social functioning and therefore should be addressed in social skills interventions. Ongoing pursuits aimed at elucidating etiological factors contributing to the notable impairment in face processing in ASD may be instrumental in guiding early remediation with far-reaching developmental outcomes. While the current study examined the predictability of social perception on social functioning, the reverse is also highly plausible and such investigations are needed. Finally, the study of social ability in ASD requires multiple levels of analysis to include social brain networks, cognitive processes, social behavior, and ultimately examination of functioning across time, people, and contexts [2].

## Conclusions

Social functioning is complex and is comprised of many interwoven constructs. To our knowledge, this is the first study to investigate the association between the neuropsychological assessment of facial information (affect, identity, and memory) and social functioning measured explicitly in social interaction with peers. Moreover, the study of behavior within the natural playground context infers social functioning in the real world. Face memory has direct effects on social interactions with peers during everyday life, which is evident for both children with ASD as well as for TD children. The findings not only contribute to the literature showing that children with ASD exhibit impairment in face perception and especially face memory but also demonstrate that this deficit is closely linked to reciprocal social functioning. Thus, the ability to identify and remember faces has a significant impact on social interaction skills. The results suggest that face memory is a core component of social interaction skills that has both the capacity to facilitate and debilitate depending on the capability of the individual.

## Competing interests

The authors declare that they have no competing interests.

## Authors’ contributions

BAC was the main contributor to the study design, statistics, and initial drafting of the manuscript. CN performed a majority of the diagnostic and neuropsychological assessments for the study. APK participated in the face processing protocol and contributed to drafting and interpreting the statistics for the manuscript. LRQ provided significant contributions to the ‘Background’ section of the manuscript and coded many of the playground behavioral videos. EKE also contributed to the drafting of the manuscript, ran playground protocols, and participated in the reliability testing for the behavioral coding. All authors read and approved the final manuscript.
